# Draft Genome Sequences of Potentially Pathogenic Clostridium perfringens Strains from Environmental Surface Water in the North West Province of South Africa

**DOI:** 10.1128/MRA.00407-19

**Published:** 2019-08-08

**Authors:** Johannes Cornelius Jacobus Fourie, Tomasz J. Sanko, Cornelius Carlos Bezuidenhout, Charlotte Mienie, Rasheed Adegbola Adeleke

**Affiliations:** a Unit for Environmental Sciences and Management, North-West University, Potchefstroom, South Africa; b Agricultural Research Council, Institute for Soil, Climate and Water, Pretoria, South Africa; Indiana University, Bloomington

## Abstract

Surface water systems in South Africa are experiencing a major decline in quality due to various anthropogenic factors. This poses a possible health risk for humans. Here, we present the draft genome sequences of three Clostridium perfringens isolates obtained from a fecally polluted river system in the North West province of South Africa.

## ANNOUNCEMENT

Clostridium perfringens is a Gram-positive bacterium that requires strict anaerobic conditions to grow. Its ability to produce endospores ensures its survival under unfavorable conditions, e.g., in aerobic environments. Due to its ubiquitous nature (especially in soil and aquatic systems), C. perfringens could also be a looming clinical problem. This species can cause severe disease in humans (1, 2). C. perfringens strains are classified into seven types (A through G) according to the production of six major toxins (alpha-, beta-, epsilon-, iota-, Clostridium perfringens enterotoxin [CPE], and necrotic enteritis B-like [NetB] toxins) (3). C. perfringens type A strains are known to cause gas gangrene (clostridial myonecrosis) and necrotic enteritis, as well as mild diarrhea, in humans (4).

This paper presents the draft genome assemblies of C. perfringens strains derived from river water during a warm rainy season. The water quality is influenced by various anthropogenic activities, including mining (gold and diamonds), agriculture, and, in particular, return flows from wastewater treatment plants (5, 6). Recently, high levels of indicator bacteria showed occurrences of fecal contamination, and various points in this river were designated potential “hot spots” for outbreaks of bacterial diseases (7; http://www.dwa.gov.za/iwqs/microbio/nmmp.asp).

Clostridium perfringens were isolated from river water using a modified version of the Fung double-tube method (8). The bacteria were grown in tryptose sulfite cycloserine agar (Oxoid, UK) at 42°C for 6 h. Single colonies were incubated anaerobically overnight in reinforced clostridial medium (Oxoid, UK) and then pelleted. Total genomic DNA was extracted from each pellet with the use of a NucleoSpin tissue kit (Macherey-Nagel). Amplification and sequencing of the 16S rRNA genes confirmed the identities of the three isolates to be C. perfringens. Paired-end sequencing libraries were generated with a Nextera XT DNA library prep kit (Illumina, San Diego, CA, USA), and this was followed by whole-genome sequencing with a MiSeq reagent kit v3 (600 cycles). Quality evaluation and trimming of short (less than 50 bp) or low-quality nucleotides (Q < 15) were performed in Trimmomatic (v.0.36) (9). *De novo* assembly was conducted in SPAdes (v.3.9.0) (10), followed by gene prediction and annotation using the NCBI Prokaryotic Genome Annotation Pipeline (v.4.3) (11). BLASTx comparison was used to search databases for virulence factors (VF) and antibiotic resistance genes (ARGs) in deepARG (v.2.0) (12, 13). Average nucleotide identity (ANI) was determined by OrthoANI (v.1.4) (14). Default parameters were used for all software unless otherwise specified.

*In silico* analysis of the C. perfringens strains (Table 1) created, on average, 110 to 205 scaffolds, with an overall average genome coverage of 186×. Draft genomes were generated with a total length of between 3.44 Mbp and 3.6 Mbp and an average G+C content of 28.18%.

**TABLE 1:** Genome characteristics and accession numbers of C. perfringens strains

Characteristic	Data for strain:		
	SC4-C13	SC4-C17	SC4-C24
No. of reads	8,746,682	8,055,822	11,557,934
Avg read length (bp)	233.678	238.49	244.97
No. of scaffolds	205	205	110
Largest contig size (bp)	1,386,217	1,397,833	1,428,470
*N*_50_	461,812	461,812	356,343
G+C content (%)	28.19	28.14	28.21
Gene annotation data			
Genome size (bp)	3,604,770	3,514,948	3,437,837
No. of CDS^*a*^	3,245	3,201	3,079
No. of total RNAs	125	124	130
No. of total rRNAs	29	29	34
No. of total tRNAs	92	91	92
No. of pseudogenes	55	66	55
GenBank accession no.	RQNR00000000	RQNQ00000000	RQNP00000000
SRA accession no.	SRR8867692	SRR8867693	SRR8867691

a CDS, coding sequences.

The draft genomes described here were also analyzed for the presence of VF and ARGs. This revealed 35 genes that encode VF such as hemolysins, enterotoxins, sialidase, collagenase, perfringolysin O, and alpha-clostripain. The genome assembly also revealed the presence of four hyaluronidase genes, as well as two members of the double-component VirR/VirS regulon. The ARG analysis revealed the presence of macrolide-lincosamide-streptogramin, β-lactam, trimethoprim, tetracycline, kasugamycin, and bacitracin genes. They also harbored the *vanRI* and *vanRG* genes, which encode glycopeptides, and *vgaB*, *arlR*, and *MepA*, which are responsible for multidrug-resistant efflux pumps.

Genomic comparison with the well-characterized C. perfringens strain 13 (GenBank accession number BA000016) resulted in values of between 98.50% and 98.52%. Therefore, our three C. perfringens strains can be classified as type A strains, which are human pathogens.

### Data availability.

These draft genome assemblies have been deposited at GenBank under the accession numbers RQNR00000000 (Clostridium perfringens SC4-C13), RQNQ00000000 (Clostridium perfringens SC4-C17), and RQNP00000000 (Clostridium perfringens SC4-C24). The Sequence Read Archive accession numbers are SRR8867692, SRR8867693, and SRR8867691, respectively.
